# Harnessing Multisensory Perception for the Tomato Agrifood Chain

**DOI:** 10.3390/s26134195

**Published:** 2026-07-02

**Authors:** Jun-Wei Liang, Yi-Jia Chen, Peng-Xian Zhang, Yun-Lang Feng, Douglas Fernandes Barbin, Wen-Hao Su

**Affiliations:** 1 College of Engineering, China Agricultural University, 17 Qinghua East Road, Haidian, Beijing 100083, China; joonway@cau.edu.cn (J.-W.L.); chenyijia@cau.edu.cn (Y.-J.C.); zpx070615@cau.edu.cn (P.-X.Z.); feng.y.l@cau.edu.cn (Y.-L.F.); 2 School of Food Engineering, University of Campinas (UNICAMP), Campinas 13083-862, SP, Brazil; dfbarbin@unicamp.br

**Keywords:** tomato, multisensory perception, digital fingerprint, multimodal fusion

## Abstract

Structural inefficiencies and labor shortages within the global tomato agrifood chain pose significant threats to its economic sustainability. While vision-dominated systems, encompassing structural and spectral dimensions, have pioneered intelligent management, their limitations in environmental robustness and computational overhead necessitate a new approach. Furthermore, dimensional incompleteness remains a challenge in decoding internal states. Multisensory perception, integrating physical (tactile and auditory) and chemical (olfactory and gustatory) modalities, enables the quantitative characterization of tomato physiological states. Based on technological advancements at the leading edge of knowledge, a critical perspective on the mushrooming field of multisensory perception is highlighted. Grounded in the capabilities and bottlenecks of visual perception, the discussion outlines significant progress in multisensory perception, along with its challenges and prospects. Crucially, it delineates the construction pathway of digital fingerprints that couple instrumental sensing signals with human sensory experiences and envisions the landscape of multimodal fusion to address practical challenges. This perspective provides a roadmap for sensorially transparent evaluation systems in the tomato agrifood chain.

## 1. Introduction

Tomatoes represent a cornerstone of the global horticultural industry, with annual production exceeding 192 million tons and a market valuation projected to reach $1691 billion by 2025 at a 7.2% compound annual growth rate [1]. Despite their immense economic scale, the industry suffers from severe inefficiencies, with post-harvest losses alone accounting for 13–17% of total production [2]. These losses stem primarily from a lack of timely and actionable quality information at critical junctures across the breeding, cultivation, harvesting, and processing stages [3]. Driven by the escalating global pressures of population aging and structural labor shortages, there is an urgent necessity to transition the entire tomato agrifood chain toward intelligent and automated management [4].

Over the past decade, optical sensing technologies, encompassing machine vision, near-infrared (NIR) spectroscopy and hyperspectral imaging (HSI), have driven this intelligent transition, establishing themselves as the dominant approaches for non-destructive quality assessment. During the cultivation phase, drone-mounted multispectral and hyperspectral systems effectively monitor canopy leaf nitrogen content to optimize fertilizer application, achieving high predictive fidelity (*R*^2^ of 0.88) [1]. Furthermore, post-harvest ripeness classification has been significantly enhanced through RGB-NIR fusion frameworks, reaching 94.8% accuracy and decisively outperforming single-modal optical baselines [2].

However, as these technologies scale, the inherent limitations of vision-dominated perception are becoming critically apparent. First, due to dimensional incompleteness, it is incapable of directly capturing crucial quality attributes such as texture firmness, internal acoustic resonances or volatile flavor profiles. Second, environmental robustness remains a persistent challenge [3]. Under fluctuating lighting and foliage occlusion, conventional recognition systems frequently exhibit misclassification rates ranging from 15% to 27% [2]. Third, the substantial computational overhead required to process massive amounts of redundant data generates severe latency, structurally hindering real-time inference and edge-deployment capabilities [3].

To address these bottlenecks, the perception paradigm is accelerating toward multisensory fusion. Comprehensive tomato quality inspection inherently requires the synthesis of multiple sensory dimensions. Tactile modalities utilizing flexible soft grippers can safely evaluate surface hardness and elasticity, achieving robust ripeness grading accuracy (84.6%) without inflicting mechanical damage [5]. Simultaneously, acoustic sensors detect internal wave velocity changes, while electronic noses and tongues quantitatively analyze volatile organic compounds and dissolved flavor chemicals [3]. Furthermore, the application of digital fingerprinting establishes a profound modeling connection that maps raw sensing signals to human sensory experiences. Formally, a digital fingerprint is defined as a multidimensional, quantitative representation generated by mapping high-dimensional, heterogeneous instrumental sensor data to human subjective sensory metrics through mathematical dimensionality reduction and predictive modeling. By decoding physical and chemical cues, the system effectively translates these multidimensional signals into intuitive sensory descriptors [6]. Building upon this foundation, multimodal fusion architectures demonstrate exceptional perception performance via cross-modal complementarity [7].

While not formulated as a quantitative systematic review, this perspective draws upon representative literature primarily sourced from authoritative databases, including Web of Science, Elsevier, and IEEE Xplore. To ensure a comprehensive evaluation of current advancements, we employed a hybrid search strategy combining Boolean logic and semantic retrieval. The literature retrieval specifically focused on a defined time window (2021–2026). The core keyword matrix encompassed the target crop (“tomato”) alongside multisensory dimensions (“visual”, “spectral”, “tactile”, “acoustic”, “gustatory”, “olfactory”) and relevant methodologies (e.g., “multimodal fusion”).

This perspective delineates a paradigm shift in agrifood informatics by contextualizing advanced computational concepts within the tomato agrifood chain. First, we transition from generic multimodal fusion to a specialized agricultural multisensory framework. By synthesizing visual, tactile, auditory, olfactory, and gustatory inputs, we frame digital fingerprints that map high-dimensional machine signals to subjective sensory metrics, effectively quantifying human-like quality perception. Second, we conceptualize the agricultural digital twin as an information totality—a multidimensional, self-optimizing engine integrating heterogeneous signals across breeding, cultivation, harvesting, and processing to mirror the crop’s holistic state and facilitate closed-loop decision-making. Third, we address the practicalities of unstructured agricultural environments, uneven technological readiness, and edge-deployment constraints by proposing constraint-driven fusion frameworks that diverge from idealized, server-centric models. Collectively, these elements redefine agricultural intelligence, envisioning robotic systems that surpass traditional capabilities by harmonizing physical interaction with a profound, quantified understanding of both physiological states and sensory expectations.

To systematically articulate this paradigm shift, the remainder of this paper is structured as follows: Section 2 examines the capabilities and fundamental bottlenecks of visual perception, detailing its spatial and spectral extensions (Section 2.1 and Section 2.2) while critically addressing its limitations in environmental robustness and computational overhead (Section 2.3). Section 3 explores emerging modalities beyond vision, categorizing them into physical (Section 3.1) and chemical perception (Section 3.2), and details the construction of digital fingerprints (Section 3.3). Finally, Section 4 confronts the practical challenges of sensor fusion, proposing three core strategic frameworks: scenario-driven mapping (Section 4.1), readiness-based integration (Section 4.2), and resource-aware edge deployment (Section 4.3). Ultimately, this perspective provides a clear roadmap for the sustainable development of the tomato agrifood chain, orchestrated by holistic multisensory perception.

## 2. Capabilities and Bottlenecks of Visual Perception

**Figure 1:**
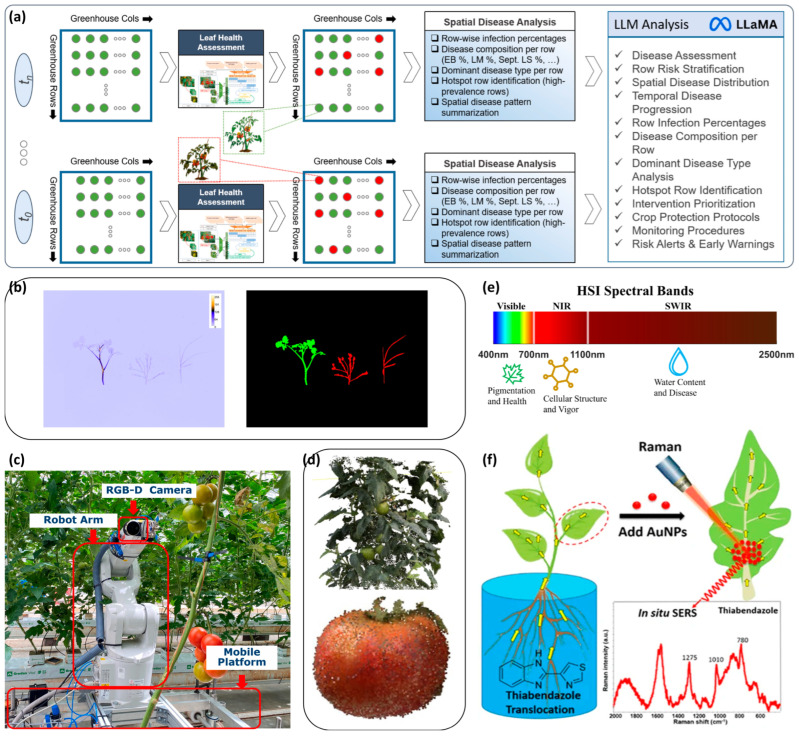
Key advancements, scientific principles, and technologies of visual modalities. (**a**) LLM-integrated pipeline for spatial disease analysis and decision-making; (**b**) Material–intelligence active visual construction: active recognition of weeds and crops using fluorescent tracer technology; (**c**) hardware setup for 3D machine vision perception of tomatoes; (**d**) 3D visual imaging of tomato plants and fruits; (**e**) mapping of physiological and biological features across different wavelength regions: visible bands support pigment-based analysis, the NIR region reveals cellular-level information indicating plant health, and the short-wave infrared (SWIR) region is utilized for assessing water content; (**f**) a surface-enhanced Raman spectroscopy (SERS) framework for the real-time tracking of systemic pesticide translocation within microscopic plant tissues. Part (**a**) is adapted from Shafay et al. [8], licensed under CC-BY 4.0 (https://creativecommons.org/licenses/by/4.0/, accessed on 29 June 2026). Part (**b**) draws on our team’s previous related research [9]. Part (**c**) is adapted from Wang et al. [10], licensed under CC-BY 4.0 (https://creativecommons.org/licenses/by/4.0/, accessed on 29 June 2026). Parts (**d**,**e**) are adapted from Karukayil et al. [11], licensed under CC-BY 4.0 (https://creativecommons.org/licenses/by/4.0/, accessed on 29 June 2026). Part (**f**) is adapted from Yang et al. [12], with permission from the Copyright Clearance Center.

Unlike conventional machine vision perception centered on object detection and localization, agricultural visual perception necessitates a dual-capability architecture: the precise parsing of spatial–morphological structures and the non-destructive decoding of internal states. This specialized requirement demands a shift from optical imaging to data inversion. By synthesizing 2D or 3D spatial imaging with spectral analysis, the perception system transcends the limitations of human sight, achieving quantitative vision. Such an evolution, moving from surface observation to physiological characterization, serves as the core technological cornerstone for the intelligent transformation of the tomato agrifood chain. Figure 1 demonstrates the key advancements, scientific principles and technologies of visual perception.

### 2.1. Spatial Expansion of Machine Vision

As the most widely applied sensing technology in the tomato industry, machine vision captures planar and spatial information to facilitate automated decision-making across the agricultural lifecycle. Figure 1 illustrates the spatial expansion of machine vision, encompassing the technological transition from 2D algorithmic enhancements and active visual construction to 3D digital twin integration.

2D vision, utilizing grayscale and RGB images, provides a ubiquitous and cost-effective approach. Its application spans the entire tomato agrifood chain, enabling full lifecycle management from seedling monitoring to post-harvest sorting. Historically, advancements in 2D visual analytics have predominantly relied on algorithmic enhancements to extract features from complex environments. In phenotyping and growth monitoring, Gu et al. [13] integrated a CASA structure into an improved RT-DETR model to automate fruit parameter extraction under complex field shading, while Zhang et al. [14] utilized a CNN–Transformer hybrid temporal network for the non-destructive estimation of the nitrogen nutrient index via 2D canopy images. During the harvesting and quality evaluation stages, deep learning continues to optimize perception: fine-tuned ResNet50 models have been deployed on large imbalanced datasets to distinguish true external defects from natural physiological structures [15]; the SegLoRA framework provides computationally efficient maturity segmentation under fluctuating greenhouse lighting; and improved Swin Transformer V2 models, combined with morphological thinning, achieve precise pedicel localization for robotic harvesting [16]. In disease control, recent frameworks have begun aligning 2D visual features with textual semantics, introducing large language models to simulate expert diagnostic reasoning for actionable plant protection [8].

While conventional approaches continuously escalate algorithmic complexity to combat background interference, cross-disciplinary innovations are enabling a significant shift from passive algorithmic processing to active visual construction. In a material–intelligence integration paradigm, Zhang et al. [9] actively constructed target fingerprints to bypass the computationally intensive deep neural networks traditionally required for weed–crop differentiation. By pre-treating tomato roots with fluorescent tracers (sodium fluorescein and rhodamine B), systemic physiological translocation concentrates these compounds in vascular tissues, generating a machine-readable signal distinct from chlorophyll fluorescence. This biochemical approach inherently resolves the confusion caused by morphologically identical items, reducing complex semantic segmentation to millisecond-level optical thresholding. This demonstrates how cross-disciplinary innovations can eliminate the perceptual system’s reliance on high-performance computing units.

Building upon 2D capabilities, 3D vision reconstructs spatial geometries to overcome scale distortion and depth ambiguity, significantly improving target isolation in dense canopies. In spatial phenotyping, Zhu et al. [17] developed a multi-view depth sensing framework utilizing 3D-NDT and DBSCAN algorithms for precise stem–leaf separation and morphological parameter extraction. Advancing toward cyber–physical systems, Lang et al. [18] proposed a digital twin-driven harvesting framework. By mapping 3D point clouds into a high-fidelity virtual greenhouse in the Unity engine, this approach leverages reinforcement learning to globally optimize robotic docking, trajectory planning, and hand–eye coordination.

Despite these remarkable advancements, both 2D and 3D optical vision systems face inherent bottlenecks: they struggle to penetrate severe foliage occlusion and are fundamentally incapable of assessing internal biochemical compositions. To achieve non-destructive internal quality testing, spectral technology has emerged as a mandatory extension within the overall perceptual system.

### 2.2. Spectral Extension for Internal Quality

To overcome the inherent inability of spatial vision to analyze internal physiological structures, spectroscopic technology leverages light–matter interactions (absorption, reflection, transmission, and scattering) to quantitatively invert the molecular composition of fruits. It enables non-destructive, non-contact evaluation, elevating visual perception systems from superficial phenotypic observations to the quantitative analysis of internal quality attributes specific to tomatoes, such as soluble solids, acidity, and health-promoting antioxidants.

As a robust non-destructive modality, Vis-NIR spectroscopy and multispectral imaging drive the transition from reactive visual inspection to proactive spectral diagnosis, particularly in pre-symptomatic pathogen monitoring and dynamic biophysical characterization within the tomato industry. For early disease control, specific spectral biomarkers combined with Relief feature selection and machine learning algorithms (e.g., SVM, kNN) enable the pre-symptomatic detection of *Clavibacter michiganensis* subsp. *michiganensis* (a devastating tomato bacterial canker) with up to 100% accuracy days before foliar symptoms manifest [19]. Similarly, to quantify pest stress on tomato foliage, specific water absorption bands (1450 and 1900 nm) serve as precise indicators positively correlated with leaf miner infestation severity [20], while in post-harvest scenarios, Vis-NIR coupled with neural networks achieves 96% accuracy in detecting *Rhizopus stolonifer* conidia, effectively mitigating cross-contamination rot risks in packaged red tomatoes [21]. Beyond pathology, this modality is pivotal for in situ quality estimation under fluctuating greenhouse illumination. By utilizing dynamic calibration methods alongside partial least squares (PLS) regression, the system effectively filters environmental noise to yield laboratory-grade predictions for the soluble solid content of tomatoes directly on the vine (*R*^2^ = 0.95, RMSEP = 0.29 °Brix) [22]. Scaling this perceptual capability to the canopy level, the fusion of high-resolution multispectral imagery with terrestrial LiDAR point clouds resolves structural ambiguities within the dense tomato canopy, facilitating highly accurate (92%) crop discrimination and advanced biophysical tracking of crown area and biomass across varying nitrogen nutrient levels [23].

Expanding beyond broad-band Vis-NIR, HSI acquires a three-dimensional spatial–spectral data cube across hundreds of narrow bands, offering diagnostic granularity for complex physiological stresses and multi-attribute tomato quality monitoring. Under controlled soilless tomato cultivation, HSI cameras can detect water deficit severity as early as the first day of withheld irrigation, where strategic threshold shifts in specific indices (e.g., a 16% increase in TCARI) accurately mirror leaf chlorophyll depletion and substrate volumetric water drops [24]. Similarly, extended-range HSI establishes robust decision-supporting classification for salinity stress (specificity = 0.94), enabling the precise, non-destructive quantification of toxic ion accumulation in grafted tomato plants [25]. Advancing to physiological resource allocation, portable spectroradiometers coupled with PLS algorithms quantitatively map sucrose concentrations in tomato petiole ends with exceptional precision (*R_P_*^2^ = 0.98, RPD = 7.12) [26]. This spectral-metabolic mapping precisely identifies occluded senescent leaves characterized by negative net assimilates, providing a theoretical basis for intelligent tomato canopy pruning. On the reproductive front, the immense data dimensionality of HSI is being strategically distilled to formulate cost-effective, pre-harvest fruit quality monitoring devices. By utilizing a hotspot overlapping method to select merely five optimal bands, machine learning models can simultaneously predict seven distinct biophysical and biochemical parameters of tomatoes, achieving remarkable predictive fidelities for fruit weight (*R*^2^ = 0.95), firmness (*R*^2^ = 0.89), lycopene (*R*^2^ = 0.85), and total soluble solids (*R*^2^ = 0.72) [27].

Complementing the macroscopic biophysical capabilities of Vis-NIR and HSI, Raman spectroscopy leverages the inelastic scattering effect to capture highly specific vibrational and rotational molecular fingerprints, offering unparalleled precision for trace metabolite detection due to its inherent immunity to water absorption—a critical advantage for high-moisture tomato fruits. In the realm of plant protection, Raman coupled with chemometrics successfully discriminates devastating tomato viral pathogens like TYLCSV and TSWV with over 85% accuracy merely 8 days post-inoculation [28]. At the micro-physiological level, SERS enables the real-time spatial mapping of systemic pesticide (e.g., thiabendazole) translocation from hydroponic tomato roots through leaf midribs to trichomes. Crucially, SERS identifies specific molecular toxicity responses, such as the emergence of an adenine-related peak at 737 cm^−1^, serving as a novel dynamic biomarker for pesticide-induced stress [12]. For the precise quantification of internal quality, excitation wavelength optimization is paramount: while 532 nm excitation effectively captures qualitative compositional shifts from lycopene to *β*-carotene, the 785 nm wavelength delivers the most robust quantitative carotenoid models in tomatoes [29]. Progressing from single-point analysis to spatial visualization, Raman chemical imaging accurately maps internal lycopene distributions across various post-harvest tomato ripening stages. Pushing the boundaries of non-destructive assessment, the pioneering application of spatially offset Raman spectroscopy successfully retrieves subsurface spectral signatures through the tomato pericarp, laying a definitive foundation for the true volumetric evaluation of internal fruit maturity without tissue disruption [30].

Looking forward, the intelligent upgrading of the tomato agrifood chain is driven by three key trajectories. First, spectral sensors are evolving toward miniaturization, facilitating their deep integration into automated robotic end-effectors for real-time, on-the-fly quality assessment during greenhouse tomato harvesting. Second, multi-source data fusion, combining multispectral signatures with spatial vision, is being deployed to enhance target localization and minimize mechanical damage to the delicate tomato epidermis in complex agronomic environments. Ultimately, these perceptual technologies are transitioning from isolated post-harvest scenarios to comprehensive, full-lifecycle deployments spanning targeted breeding, precision cultivation, and automated harvesting, thereby fully unlocking the physiological and commercial potential of the tomato industry.

### 2.3. Environmental Robustness and Computational Overhead

While the integration of spatial machine vision and spectral modalities provides full-dimensional coverage—spanning external phenotypes to internal biochemical quality—this optical-centric perception faces critical bottlenecks that hinder its scalable deployment across the tomato agrifood chain. Foremost, optical perception is intrinsically vulnerable to environmental stochasticity. Because detection accuracy relies heavily on stable illumination and consistent optical signal acquisition, the dynamic and unpredictable nature of real-world agricultural scenarios introduces severe interference. These environmental fluctuations inevitably degrade image fidelity and disrupt signal integrity, frequently resulting in target feature extraction failures and erratic detection performance.

To mitigate this, front-end low-level image restoration has emerged as a crucial prerequisite before executing downstream recognition tasks. Recent state-of-the-art advancements, such as the Real2Real invariant style alignment framework, have demonstrated remarkable efficacy in addressing complex real-world sensor noise through unsupervised one-shot denoising [31]. By effectively restoring signal fidelity at the foundational level, these preprocessing strategies provide essential robustness against environmental interference.

However, deploying these sophisticated low-level restoration algorithms inherently imposes an initial computational tax on the perception system. Furthermore, while such processing successfully suppresses noise and illumination artifacts, it fundamentally cannot overcome the intrinsic physical boundaries of optical modalities—most notably, severe physical occlusion within dense canopies or the inability to penetrate deep internal tissues. Crucially, attempting to circumvent these inherent physical limitations merely by stacking increasingly profound visual neural networks or integrating ultra-high-dimensional optical data is hardly a feasible or sustainable solution.

This relentless pursuit of visual perceptual enhancement inadvertently exacerbates an exponential explosion in data dimensionality. High-density 3D point clouds, hyperspectral data cubes, and high-resolution Raman spectra impose prohibitive computational and storage demands. Consequently, deploying these data-intensive architectures in resource-constrained edge environments remains a formidable challenge. Although lightweight model compression is a ubiquitous workaround for edge deployment, it inevitably sacrifices crucial feature dimensions and diagnostic accuracy, thereby severely bottlenecking the large-scale industrial application of these advanced perception systems.

## 3. Beyond Vision: Emerging Multisensory Modalities

Introducing multisensory perception offers a promising pathway to mitigate the dimensionality constraints, environmental vulnerabilities, and computational bottlenecks of conventional vision and spectral inversion. Crucially, the transition from visual limitations to multisensory solutions is not a generic upgrade, but a targeted mapping of specific remedies to distinct perceptual bottlenecks. For instance, tactile sensing circumvents the severe computational overhead and occlusion vulnerabilities of complex 3D visual reconstruction by directly quantifying macroscopic mechanical properties (e.g., firmness and elasticity) through physical contact. Auditory perception overcomes lighting dependencies and visual obstructions by capturing acoustic emissions that signal hidden physiological stress long before visible symptoms appear. Meanwhile, chemical perception bridges the dimensional incompleteness of surface imaging by directly decoding internal trace metabolites, eliminating the reliance on computationally expensive and environment-sensitive hyperspectral setups. Through this precise allocation of sensing modalities, the system achieves sensory transparency—formally defined as the capability to quantitatively and transparently decode agricultural physiological states that directly correlate with human sensory experiences. By establishing this transparency, these targeted modalities help to bridge the gap between equipment and humans, adding critical value to comprehensive quality assessment.

Crucially, the integration of these sensory modalities must be strictly task-driven rather than the blind stacking of sensors. Specific multisensory elements are selectively deployed to resolve the exact physiological states that visual perception fails to efficiently capture. By strategically integrating visual, tactile, auditory, olfactory, and gustatory data, a centralized, multidimensional digital twin model of the agricultural entity is constructed. This perception core is then seamlessly applied across the four chronological stages of the agrifood chain: seed breeding, smart cultivation, autonomous harvesting, and precise processing.

**Figure 2:**
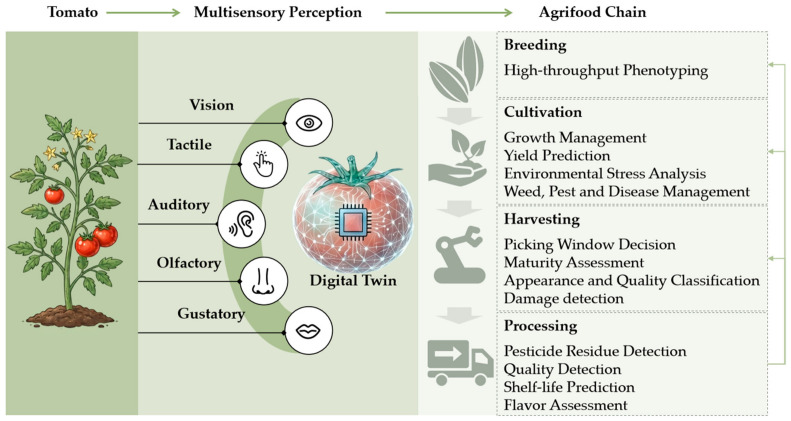
Multisensory perception and application architecture across the tomato agrifood chain. By integrating the five sensory modalities of a tomato—vision, tactile, auditory, olfactory and gustatory—a digital twin model mapping the entity can be constructed. This centralized multidimensional perception core is subsequently applied across four critical stages of the agrifood chain: seed breeding, smart cultivation, autonomous harvesting and precise processing. The inner arrows illustrate the chronological flow across the sequential stages of the agrifood chain, while the outer arrows represent the reverse feedback mechanism, routing downstream sensory data from the processing stage back to optimize upstream harvesting decisions, cultivation management and breeding strategies to establish a fully closed-loop ecosystem.

Looking forward, overcoming the isolated data silos typical of conventional linear agricultural chains requires a shift toward a digital twin-driven closed-loop feedback mechanism. Within this envisioned framework, terminal sensory evaluations from the downstream processing stage would no longer be restricted to passive, end-of-line quality grading. Instead, by utilizing a unified data ontology, we propose structuring these multimodal evaluations into standardized, quantifiable feedback parameters. These multidimensional parameters can then be reverse-fed through a cloud architecture to systematically optimize upstream operational nodes. In a fully realized deployment, downstream firmness and metabolic profiles could continuously calibrate optimal picking windows during autonomous harvesting; trace physiological stress indicators would guide the dynamic adjustment of management during cultivation; and holistic sensory traits would ultimately inform precise phenotyping for targeted genetic breeding. By conceptually closing the loop from post-harvest evaluation back to pre-planting decisions, this proposed architecture provides a blueprint for an intrinsically data-driven adaptive agricultural ecosystem (Figure 2).

**Figure 3:**
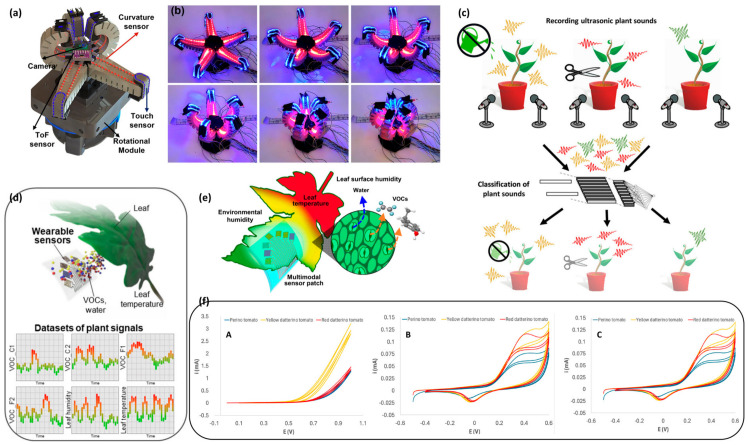
Representative advancements in emerging multisensory modalities. (**a**,**b**) Tactile-enhanced soft robotic gripper. (**a**) CAD design highlighting integrated tactile and curvature sensors, alongside visual and time of flight (ToF) modules. (**b**) Various grasping states demonstrating tactile and curvature sensing via embedded optical waveguides. (**c**) Acoustic perception of plant stress. Airborne ultrasonic sounds emitted by stressed plants (e.g., dehydrated or injured) are recorded and classified via machine learning to identify their physiological conditions. (**d**,**e**) Wearable multimodal leaf sensors. Schematic of a sensor patch attached to the abaxial leaf surface for real-time monitoring of volatile organic compounds (VOCs), water emissions, and temperature, coupled with machine learning for signal classification. (**f**) E-tongue signal profiles. Cyclic voltammetry responses of different tomato puree samples using various modified electrodes, where A, B, and C correspond to copper nanoparticles, gold nanoparticles and poly(3,4-ethylenedioxythiophene)-modified electrodes, respectively. Parts (**a**,**b**) are adapted from Mishra et al. [32], licensed under CC-BY 4.0 (https://creativecommons.org/licenses/by/4.0/, accessed on 29 June 2026). Part (**c**) is adapted from Khait et al. [33], licensed under CC-BY 4.0 (https://creativecommons.org/licenses/by/4.0/, accessed on 29 June 2026). Parts (**d**,**e**) are adapted from Lee et al. [34], licensed under CC-BY-NC 4.0 (https://creativecommons.org/licenses/by-nc/4.0/, accessed on 29 June 2026). Part (**f**) is adapted from Magnani et al., licensed under CC-BY 4.0 (https://creativecommons.org/licenses/by/4.0/, accessed on 29 June 2026) [35].

While the coordination of multisensory modalities enhances perception dimensions, it is often accompanied by increased costs in hardware architecture and data processing. However, economic viability is not inherently hindered by all modalities. Standard visual and auditory sensors demonstrate high economic feasibility for real-time deployment. Meanwhile, tactile and olfactory modalities present medium economic feasibility. In contrast, gustatory sensors and spectral imaging involve higher capital or computational costs, resulting in lower economic feasibility. In practice, high-precision (spectral and gustatory) modalities are typically utilized for benchmarking and calibration, while cost-effective sensors serve as the primary modules for in situ deployment. The sensor-as-a-service model can alleviate the burden of capital-intensive ownership associated with high-precision instruments. Furthermore, by establishing cross-modal mappings between in situ sensors and high-end benchmarks, the framework can utilize high-precision proxy models. Such a balanced configuration allows the system to cater to both mass-market and high-value quality-oriented segments. Consequently, precise sensory data interventions can help mitigate waste across the chain and enhance the added value of product grading, thus offering possibilities for increased overall revenue. Seeking a balance between configuration costs and potential benefits represents a crucial prerequisite for driving the large-scale deployment of this technology. Through rational spatial and temporal configuration, this initial investment holds the potential to translate into tangible economic returns. Figure 3 demonstrates the representative advancements in emerging non-visual multisensory modalities.

### 3.1. Physical Perception: Tactile and Auditory Modalities

Within the tomato agrifood chain, the implementation forms and application scenarios of tactile and auditory perception rely on purely physical signals. Consequently, the acquisition and mathematical modeling of parameters—such as force–displacement curves and acoustic waveforms—are relatively straightforward.

Inspired by the human somatosensory system, where specialized mechanoreceptors perceive vibration, pressure, and texture, robotic tactile sensors translate physical interactions into electrical signals. Based on their transduction principles, these sensors are primarily classified into five categories (Table 1). To optimize operational efficiency across the tomato agrifood chain, these sensing mechanisms must be strategically deployed according to their specific physical advantages and limitations. Specifically, piezoresistive sensors, leveraging their high sensitivity and strong overload capacity, are well suited for the initial robotic grasping of tomatoes, where detecting delicate contact while withstanding sudden mechanical overloads is critical. Capacitive sensors, characterized by their good linearity and excellent real-time performance, are optimally deployed in high-speed firmness sorting lines. Piezoelectric sensors, featuring wide dynamic ranges and durability, are highly reliable for monitoring transient events, such as slip detection during automated packaging and collision impacts during logistics. Photoelectric sensors, offering high spatial resolution and immunity to electromagnetic interference, are highly valuable for stationary precision quality inspection to detect localized micro-bruises. Finally, the low manufacturing cost and wide measurement range of inductive sensors make them a practical choice for heavy-duty applications, such as bulk weight estimation in harsh post-harvest processing environments.

To replicate human-like tactile perception, recent studies have developed specialized sensors for agricultural applications. For instance, Chen et al. [36] developed a self-powered piezoelectric hydrogel sensor that utilizes differential piezoelectric responses under a mere 1 N of static pressure to accurately distinguish tomato ripening stages. Expanding on this, an electronic tactile sensory system was introduced to measure interfacial capacitance and resistance, capturing subtle differences in texture and hydration to classify plant species and growth stages [37]. Bridging the optical and mechanical domains, Meng et al. [38] proposed a tactile-proximity dual-mode photoelectric sensor. By embedding this sensor into a robotic gripper and integrating it with the TPNet model, the system achieved 94.4% accuracy in classifying tomato ripeness using only dual-mode physical feedback during grasping. Given the delicate nature of tomatoes, such tactile feedback is indispensable for mitigating impact bruising, preventing slippage, and verifying fruit–stem separation, offering the robotic system a crucial window for error recovery.

Similarly, robotic auditory perception mimics the human mechanical-to-electrical auditory pathway. In agriculture, this under-researched modality provides an irreplaceable dimension of physical information, which can be categorized by whether the plant acts as an active sound emitter or a passive acoustic medium. In active sound source-driven perception, research leverages spontaneous physiological sounds actively emitted by the plant. For example, xylem cavitation in drought-stressed tomato plants generates airborne ultrasonic emissions [39]. Captured by microphones and classified via machine learning, these signals can be integrated into wireless sensor networks to autonomously trigger precision irrigation [33]. Conversely, in passive sound source-driven perception, the plant acts as a passive medium responding to external acoustic or mechanical excitations. For instance, impact resonance techniques are extensively used to evaluate fruit hardness. By exciting a tomato with a mechanical impulse, flexible piezoelectric sensors can capture the resonant frequency to calculate the elasticity coefficient—a metric strongly correlated with traditional puncture forces and highly reliable for monitoring firmness degradation [40,41]. Furthermore, by transmitting a sine sweep acoustic signal through a tomato, researchers observed a visible decrease in signal amplitude as the fruit softens. Converting these signals into feature envelopes allows support vector machines to estimate storage duration with an average error margin of less than 0.5 days [42]. Although highly susceptible to environmental background noise, decoding the hidden correlations between plant physiological states and auditory signals remains a promising pathway for multidimensional crop monitoring.

A systematic comparison of these two acoustic paradigms reveals distinct engineering trade-offs. Active acoustic sensing offers the critical advantage of continuous and non-invasive physiological monitoring, making it highly suitable for early detection and dynamic scheduling in controlled greenhouses. However, its primary limitation lies in the ultra-low amplitude of spontaneous biological emissions, rendering it highly vulnerable to ambient agricultural noise. Conversely, passive acoustic sensing benefits from a controllable, high signal-to-noise ratio and well-established resonance physics, making it exceptionally reliable for high-speed post-harvest firmness grading and shelf-life estimation. Nevertheless, passive approaches necessitate physical excitation mechanisms and are fundamentally constrained by the target’s geometric symmetry.

Despite these advancements, purely physical perception faces inherent limitations. The sensitivity of a single mechanical parameter to ripeness is often limited and highly non-linear, and near-threshold ripeness stages show a marked decrease in signal distinguishability due to the cross-influence of multiple factors. Furthermore, tactile sensors struggle with fluctuating contact conditions, long-term sensing drift, hysteresis, and crosstalk between multi-sensor units. Meanwhile, acoustic methods strictly require surface integrity, primarily reflecting global average mechanical properties while failing to detect localized, spotty deterioration.

Furthermore, water stress in tomatoes simultaneously manifests through tactile and auditory alterations that share identical underlying biochemical drivers. The loss of cellular turgor pressure and the enzymatic depolymerization of cell wall pectin degrade the structural rigidity of the plant tissue [43]. Macroscopically, this degradation is captured by tactile modalities as a prominent reduction in the static elastic modulus—a highly robust signal, yet limited in its spatial granularity [44]. Concurrently, at the microstructural level, these cellular alterations modify the heterogeneous internal interfaces among the pericarp, locular gel and air cavities. This structural evolution induces multiple scattering and frequency-dependent energy attenuation of propagating acoustic waves [45]. While these auditory signals are highly attenuative and relatively weak, they encode high granularity regarding internal physiological configurations. This physical orthogonality establishes a complementary sensing paradigm. Fusing the reliable macroscopic baseline of tactile mechanics with the high-resolution micro-dynamics of auditory sensing helps to compensate for their respective limitations (low spatial resolution and weak signal-to-noise ratio). Consequently, this mechanical–acoustic synergy yields a granular sub-surface profile, outperforming the superficial penetration limits of conventional machine vision and spectral inversion. Crucially, this synergistic approach extends far beyond hydration monitoring, enabling robotic systems to achieve non-destructive characterization of fruit physiological states across the tomato agrifood chain.

### 3.2. Chemical Perception: Olfactory and Gustatory Modalities

While physical modalities quantify macroscopic structural properties, chemical perception interrogates the core biochemical essence of tomatoes, bridging a critical gap left by visual and spectroscopic technologies in detecting trace metabolic substances. Biomimicking human olfaction and gustation, chemical sensing systems utilize the non-specific, cross-responsive patterns of sensor arrays to construct holistic characteristic fingerprints without requiring the complex separation of individual components. Currently, it is predominantly driven by electronic nose (E-nose) and electronic tongue (E-tongue) systems. E-noses leverage gas sensor arrays to perform non-contact and rapid detection of VOCs. Conversely, E-tongues rely on electrochemical arrays modified with specific sensitive materials to quantify non-volatile liquid-phase components, providing a quantitative basis for evaluating key quality indices.

Across the tomato agrifood chain, olfactory modalities have demonstrated exceptional utility in both real-time physiological monitoring and post-harvest quality profiling. In the realm of smart cultivation, the emergence of wearable sensors—such as leaf-attachable graphene-based patches—allows for the non-invasive, real-time profiling of VOC markers. These systems can differentiate 13 individual VOCs with over 97% accuracy, enabling the early detection of late blight and mechanical damage in field environments [46]. For post-harvest evaluation, E-nose technology serves as a powerful surrogate for traditional physicochemical analysis. By coupling MOS-based sensor arrays with advanced algorithms like the extreme learning machine, researchers have successfully predicted core quality indices including acidity, soluble solids, and firmness in treated cherry tomatoes [47]. Furthermore, the synergy between HS-SPME-GC-MS/MS and E-nose enables the deep unraveling of aroma characteristics across diverse cultivars; recent models utilize these comprehensive odor fingerprints to achieve high-precision discrimination of fruit colors and varieties, thereby facilitating standardized flavor evaluation in breeding and commercial sorting [48].

In the gustatory dimension, E-tongue technology provides a multidimensional characterization of tomato taste profiles. Recent research has demonstrated that E-tongue systems can decisively discriminate between diverse cultivars at six distinct harvest maturities, with sensor responses showing significant correlations with total soluble solids, pH, and organic acids [49]. Beyond fresh fruit, gustatory modalities excel in the rapid classification of processed products. For instance, a voltammetric E-tongue modified with poly(3,4-ethylenedioxythiophene) enabled fast and effective cultivar-based classification of tomato purées, offering a low-cost solution for industrial quality control and variety traceability [35]. Although its reliance on liquid-phase contact detection requires destructive pretreatments—thereby precluding in situ and non-destructive analysis—it nevertheless remains a core quality evaluation method directly aligned with human senses. By employing representative sampling to mitigate these destructive limitations, the E-tongue can serve as a benchmark to calibrate non-destructive modalities like vision and spectroscopy. This synergy establishes a hierarchical data linkage, ensuring that perception data remains firmly tethered to core human senses, effectively bridging the disconnect between instrumental detection and subjective sensory evaluation.

Despite these promising applications, the transition of chemical sensing from controlled laboratory environments to large-scale commercial deployment is severely restricted by multidimensional technical bottlenecks. Regarding inherent sensing stability, mainstream E-noses suffer from significant baseline drift and susceptibility to ambient temperature and humidity fluctuations, while E-tongues are highly prone to electrode passivation in complex matrices. Algorithmic generalization remains a formidable obstacle. Current calibration models exhibit weak biological interpretability and poor adaptability across varying tomato cultivars and cultivation protocols. The necessity for continuous model recalibration upon switching target batches stifles the system’s ability to meet the dynamic engineering demands of agricultural environments.

In summary, while non-visual multisensory perception effectively mitigates the inherent limitations of visual systems, translating these technologies into practical field deployment and achieving broad agricultural adoption requires a rigorous evaluation of multidimensional engineering parameters. Table 2 presents a comprehensive comparison of these modalities across critical metrics. Moving forward, future research on non-visual multisensory perception must prioritize overcoming the fundamental deployment barriers by advancing targeted engineering solutions. Particular emphasis should be placed on resolving the complex integration of tactile sensing with end-effectors through miniaturized soft materials and sensor-gripper co-design, mitigating ambient field noise interference in auditory systems via directional acoustic arrays and artificial-intelligence-driven noise filtering, and significantly enhancing the environmental robustness while reducing the operational costs of olfactory and gustatory technologies by leveraging anti-interference nanomaterials and transfer-learning-based calibration models.

### 3.3. Digital Fingerprint: Mapping Instrumental Signals to Sensory Evaluation

In the modern agrifood chain, the ultimate commercial value of tomatoes is dictated by consumer sensory experience. While agricultural embodied intelligence can precisely acquire multidimensional physicochemical data, a semantic gap remains between objective instrumental signals and subjective human perception. Bridging this gap to realize quantitative, non-destructive sensory assessment requires the construction of digital fingerprints. From a computational perspective, this mapping relationship can be formalized as a predictive transfer function linking two distinct domains. The input domain represents the high-dimensional instrumental sensor space, while the target output domain denotes the multidimensional human subjective sensory evaluation space. Ultimately, the digital fingerprint acts as a robust algorithmic transformation or non-linear model that optimizes the prediction between the raw instrumental inputs and the actual human sensory ground truths. The practical execution of this concept relies on a three-stage approach to map high-dimensional sensor data to human tasting metrics:

1. Dimensionality Reduction and Feature Extraction: Raw data generated by multimodal sensors are intrinsically high-dimensional, redundant, and covariant. To distill robust digital fingerprints, mathematical transformations of the raw signals are imperative. For instance, Ghaffari et al. [55] extracted temporal features, including divergence, absorption, and desorption, from tomato VOCs captured by an E-nose. By employing principal component analysis to project the complex signals into a low-dimensional feature space, they effectively isolated healthy plants from infected ones. This provided high-quality, low-noise inputs for subsequent classification models.
2. Human Expert Panel Evaluation: Systematic human evaluation is indispensable to establish the reliable ground truth labels for machine learning models. As demonstrated by Vallverdú-Queralt et al. [56], trained sensory tasting panels utilize quantitative descriptive analysis and standardized protocols (e.g., ISO 13299:2010 [57]) to define a tomato-specific lexicon. This lexicon encompasses dimensions like freshness, green and acidulous. Calibrated against clear reference standards, this rigorous human assessment converts ambiguous personal preferences into a highly precise, standardized sensory matrix, anchoring the chemical fingerprints to actual human experience.
3. Correlation and Predictive Modeling: The final stage maps the extracted instrumental fingerprints to quantitative sensory scores. Recognizing the complex, nonlinear cross-modal interactions between sensory attributes, advanced regression and classification algorithms are deployed. Pamungkas et al. [58] exemplified this by coupling low-resolution portable Vis-NIR spectroscopy with ANNs and support vector machines (SVM). This approach successfully mapped optical signals to core sensory determinants, predicting firmness (*R*^2^ = 0.95) and total soluble solids (*R*^2^ = 0.82) with exceptionally high accuracy. The SVM classification model also achieved 96% accuracy in categorizing the maturity level. Through these algorithmic architectures, the perceptual system is effectively taught to digitally perceive and evaluate sensory attributes.

Looking forward, the application of sensory digital fingerprints must transcend generic quality sorting by establishing a direct mapping between multimodal sensor signals and the subjective culinary experience specific to tomatoes. Given that tomatoes are climacteric fruits exhibiting asynchronous ripening within a single truss, traditional phenotypic grading frequently fails to reflect true flavor maturity. By embedding these digital fingerprints into robotic harvesters and sorting lines, the industry can translate objective physicochemical signals—such as optical scattering variations in the high-moisture epidermis or specific volatile emission trajectories—directly into quantitative consumer metrics like sweetness, acidity and herbaceous aroma. This approach shift enables non-destructive, single-fruit sensory valuation, allowing the supply chain to transition from broad morphological sorting to precise flavor profiling, thereby ensuring that each tomato meets exact consumer sensory expectations and maximizing its market added value.

Despite the immense potential of digital fingerprints, their global proliferation is currently constrained by a severe lack of data standardization. Existing multimodal datasets for tomatoes are predominantly multi-source at the visual level, encompassing optical information such as visible light, depth, and near-infrared [59]. Comprehensive multisensory datasets have not yet been systematically constructed, resulting in the absence of unified supporting standards. Nevertheless, intra-modal signal non-standardization within non-visual modalities is actively occurring. The commercial hardware outputs across the four modalities exhibit structural heterogeneity. Distinct manufacturers employ proprietary baseline calibrations and unstandardized response curves, ranging from diverse dynamic pressure matrices to proprietary closed-source smell IDs. This fundamental hardware-level fragmentation inevitably creates isolated data silos, hindering global data sharing across the tomato agrifood chain. This standardization is exceptionally crucial for emerging foundation models, which depend heavily on structurally consistent data to perform accurate semantic alignment. To achieve interoperability, establishing international open-source data standards for multisensory modalities is imperative. Specifically, we outline an intra-modal standardization framework to decouple sensory data from proprietary hardware. This architecture begins with the environmental context layer, which dynamically records ambient interference factors during acquisition. It is followed by the hardware metadata layer, which defines the sensor’s transduction mechanism, dynamic range, and initial calibration baselines. Subsequently, the universal normalization layer mathematically transforms manufacturer-specific metrics into dimensionless, relative standardized scales. Ultimately, the open-source encapsulation layer serializes these normalized signals into universally readable ontologies (e.g., IEEE 21451 formats or SensorML). Adopting this framework holds the potential to dismantle hardware-specific silos. Furthermore, the entire standardization pipeline must strictly adhere to the FAIR (findable, accessible, interoperable, and reusable) data principles, ensuring that the fingerprints can be seamlessly retrieved and utilized by the global community.

While promoting product transparency and maximizing added value, this technological shift does not aim to usurp human inspection jobs. Instead, it leads to a significant occupational evolution. By delegating repetitive, labor-intensive quality sorting to robotic systems, human hands are liberated, and human involvement is elevated from mechanical physical labor to the high-level design and experiential refinement of sensory quality. This synergy redefines the experiential value of agricultural labor. It promotes an agroecosystem that prioritizes human well-being, enhances societal focus on sensory excellence, and deeply integrates the rational efficiency of technology with the emotional creativity of humanity.

## 4. Multimodal Fusion for Practical Challenges

As agricultural robotic systems transition from controlled laboratory environments to the dynamic tomato agrifood chain, they confront severe challenges in data integration. Chief among these is managing multi-dimensional tomato sensory data characterized by heterogeneity and asynchrony. Heterogeneity stems from the inherent structural disparity across modalities when profiling a tomato: vision provides high-dimensional spatial matrices, tactile and auditory sensors generate 1D continuous time-series, while olfaction and gustation yield sparse volatile arrays and complex liquid-phase biochemical signatures. Asynchrony arises from the mismatch in physical response times: the instantaneous optical capture of appearance contrasts sharply with the temporal delays inherent in mechanical contact deformation, volatile gas diffusion within the canopy microclimate and electrochemical gustatory reactions. To mitigate this temporal mismatch, software-based dynamic temporal alignment can be utilized to synchronize multi-rate sensor streams prior to fusion.

Recent advancements in multimodal architectures indicate that the optimal path lies in multi-layer and hierarchical feature fusion. Rather than relying on single-stage representations, aggregating multi-level structural and semantic features preserves fine-grained physical granularity while ensuring high-order semantic alignment [60]. Implementing this multi-layer intermediate alignment relies heavily on end-to-end Transformers with cross-attention mechanisms or graph neural networks (GNNs). Cross-attention dynamically maps asynchronous streams by treating spatial visual data of the tomato as queries to aggregate non-visual key–value pairs within a shared embedding space, thereby bypassing rigid temporal synchronization. Concurrently, GNNs model the non-linear relational topology of these heterogeneous tomato inputs, stabilizing feature propagation.

Specifically, multimodal fusion exhibits highly representative application potential across the entire tomato agrifood chain scenarios. During seed breeding, fusing visual morphological traits with chemical (olfactory and gustatory) profiles enables high-throughput screening for flavor-oriented phenotypes. In smart cultivation, combining visual canopy imaging with auditory sensing provides unprecedented early warnings for abiotic stress, triggering dynamic irrigation. For autonomous harvesting, the integration of spatial vision and tactile feedback empowers robotic end-effectors to achieve non-destructive grasping despite severe canopy occlusions [32]. Finally, in post-harvest processing, the decision-level fusion of visual surface inspection and acoustic resonance simultaneously evaluates both external defects and internal firmness, significantly improving grading accuracy and shelf-life estimation.

However, realizing these high-level fusions relies heavily on the support of comprehensive datasets. At present, open-source repositories in agricultural informatics frequently conflate multimodal with multioptical perception rather than achieving complete multisensory integration. The severe scarcity of synchronized datasets—simultaneously incorporating visual, tactile, auditory, and chemical signals—remains a critical bottleneck. As future multisensory benchmarks emerge, evaluation protocols must also rigorously evolve; beyond conventional algorithmic accuracy, models must be specifically assessed for robustness against missing modalities, inference latency under edge-computing constraints, and cross-varietal generalization. Furthermore, field deployment introduces other logistical challenges, specifically cross-modal spatial calibration under dynamic vibrations and annotation costs for training heterogeneous models. To address these barriers, targetless auto-calibration algorithms can be explored for robust spatial alignment, while self-supervised learning paradigms offer a pathway to reduce annotation dependency.

**Figure 4:**
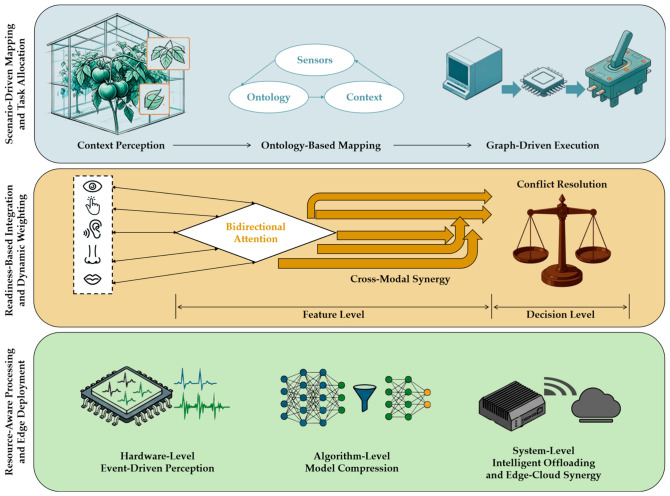
Proposed three-level multimodal fusion approach tailored for practical challenges.

Ultimately, this multi-layer multisensory convergence has the potential to establish a highly granular physiological profile of the tomato, thereby mitigating the superficial penetration limits of conventional machine vision. However, it inevitably confronts severe practical challenges [61], including sensor redundancy [62], signal asymmetry, and computational constraints at the edge [63]. To ensure the survivability of multisensory systems in real-world deployments, this chapter envisions a robust multimodal fusion architecture (Figure 4). We first comb through a scenario-driven mapping mechanism to select the minimal required sensor arrays, preventing perception overload at the source (Section 4.1). Subsequently, a readiness-based integration strategy is outlined to dynamically weight the activated modalities, resolving inter-sensor conflicts and distortions (Section 4.2). Finally, we explore resource-aware processing and edge deployment, utilizing hardware-level event-driven sensing, algorithmic model compression and system-level edge–cloud synergy to overcome local computational bottlenecks (Section 4.3).

This framework’s practical superiority becomes evident when contrasted with conventional fusion paradigms. First, while early data-level fusion preserves raw information, its vulnerability to spatial–temporal misalignment and environmental noise is mitigated by readiness-based dynamic weighting. This approach adaptively redistributes confidence based on real-time sensor reliability, resolving inter-sensor conflicts under variable occlusion. Second, intermediate feature-level fusion captures complex synergies but imposes prohibitive computational overhead. Resource-aware processing addresses this through event-driven sensing and algorithmic compression, ensuring real-time execution on mobile edge devices. Third, decision-level late fusion avoids feature complexity but blindly activates all sensors, causing perception overload. Scenario-driven mapping preempts this by autonomously parsing the operational context to trigger only essential sensor arrays. Ultimately, this three-level optimization conceptualizes a highly resilient multimodal fusion architecture tailored for unstructured agricultural applications.

### 4.1. Scenario-Driven Mapping and Task Allocation

Agricultural robots operating within the dynamic agrifood chain inherently confront a perception-computation paradox: perception overload. Traditional platforms employ an always-on architecture, simultaneously processing high-dimensional data streams from multiple sensors (e.g., LiDAR point clouds and hyperspectral cubes [11]). This indiscriminate activation not only incurs massive computational overhead but also leads to rapid battery depletion in edge devices. Such energy inefficiencies severely hinder the deployment of autonomous systems from static laboratories to real-world agrifood environments. To overcome this bottleneck, the perception approach has shifted toward a scenario-driven scheduling chain. Scenario-driven mapping can be formally defined as an adaptive resource-allocation framework where an agricultural robotic system first autonomously perceives its macro-environmental context, and subsequently queries an ontology to activate only the minimal required subset of sensors for a specific task. This mechanism optimizes computational overhead and energy efficiency by preventing perception overload at the source through the following steps:

1. Autonomous Context Perception: The foundation of this chain requires the robot to dynamically understand its macro-environment. Instead of generic processing, systems can utilize large-scale 3D semantic segmentation [64] or multi-source pseudo-label learning [65] to accurately parse unstructured agricultural scenes (e.g., distinguishing complex indeterminate tomato canopies and overlapping vines from field paths). By aggregating outputs from multiple pre-trained models to filter out misclassified labels, robots can achieve precise scene recognition even across structurally diverse target domains [65].
2. Ontology-Based Mapping: Once the operational context is identified, the system must determine the minimal necessary sensor configuration. This is typically achieved by querying ontology models like the EGO framework [66]. EGO dynamically binds specific agricultural tasks to optimal sensor subsets based on current user states, available edge resources and required accuracy. A physical blade-penetration task may solely require depth and haptic feedback, whereas biochemical quality grading prioritizes olfactory and spectral inputs [66]. This semantic mapping ensures cross-application while minimizing power consumption.
3. Graph-Driven Execution: The final phase translates these optimal mappings into sensor management. State-of-the-art approaches adopt graph-based meta-learning to implement gated switching mechanisms [67]. By utilizing graph attention networks to evaluate state awareness and system criticality in real time, the framework can dynamically toggle specific sensor branches and filters on or off. This allows the system to autonomously strike an optimal balance between safety-critical environmental awareness and power conservation in nonlinear environments [67].

Through this scheduling chain, the roboticized systems can achieve a seamless operational transition from high-light greenhouses to automated sorting lines. Rather than treating these stages as isolated silos, this architecture provides a universal perception framework across the entire tomato lifecycle. During cultivation and pre-harvest, the robot utilizes 3D semantic segmentation to parse the dense foliage and queries the EGO ontology to activate the energy-efficient sensor combination for navigation and whole-plant phenotyping. Upon transitioning to the post-harvest sorting line, the graph-based gating mechanism dynamically reconfigures the sensor array to meet the rigorous real-time demands of non-destructive internal quality assessment, substantially reducing perception overload.

Despite its efficacy, this cascading architecture remains vulnerable to the first-error effect. If severe foliage occlusion inherent in tomato vines misleads the initial scene classification, the system may load an incorrect ontology, triggering a cascade of perception failures. Moreover, the cold-start latency inherent in waking up dormant sensors poses a critical hurdle for high-speed tomato sorting lines, where millisecond-level responsiveness is non-negotiable to prevent mechanical damage to the thin-skinned fruits. Future research must prioritize highly robust, occlusion-resistant scene recognition algorithms and develop low-latency sensor activation mechanisms to ensure this framework can be reliably deployed in resource-constrained and high-speed agricultural scenarios.

### 4.2. Readiness-Based Integration and Dynamic Weighting

In the dynamic tomato agrifood chain, multimodal sensor fusion is fundamentally bottlenecked by asymmetries in technology readiness levels and signal response latencies. While optical vision provides instantaneous and high-resolution spatial data, it is susceptible to greenhouse illumination shifts. Conversely, chemical sensors can capture subtle volatile organic compounds crucial for tracking changes in tomatoes, but are plagued by significant response lags and baseline drift. This inherent heterogeneity renders static data fusion highly vulnerable, compromising both the accuracy and real-time responsiveness of robotic decision-making. To bridge this gap, we outline a readiness-based dynamic weighting framework that optimizes multimodal integration through a dual-level mechanism. Conceptually, readiness-based integration is an uncertainty-aware multimodal fusion approach that continuously evaluates the real-time reliability (i.e., technology readiness and signal integrity) of diverse sensors under fluctuating environmental conditions. By quantifying epistemic uncertainty, it dynamically redistributes confidence weights to resolve inter-sensor conflicts prior to execution. This framework operates through the following levels:

1. Feature-Level Bidirectional Attention: At the foundational level, the system must establish spatial consistency across diverse data sources. By implementing bidirectional cross-modal attention mechanisms (e.g., BAFusion) [68], the network can adaptively learn and adjust cross-modal weights. In practical greenhouse environments with dense vine occlusion or intense lighting, this mechanism autonomously suppresses distorted visual features while amplifying robust modalities, such as LiDAR-derived depth capable of penetrating complex vine structures [69]. Furthermore, integrating techniques like Monte Carlo dropout and conformal prediction provides a rigorous statistical basis for quantifying this visual distortion, ensuring highly reliable dynamic weight adjustments.
2. Decision-Level Conflict Resolution: At the higher cognitive level, the framework must manage explicit modal contradictions—for instance, when visual data indicates that a tomato is ripe based on external pigmentation, but chemical signals suggest otherwise due to abnormal internal volatile emissions (e.g., early latent decay). To resolve this, each modality first quantifies its epistemic uncertainty using the CP framework [70]. Subsequently, by applying evidential deep learning guided by Dempster–Shafer theory (D-S), the system mathematically models these uncertainty bounds and redistributes confidence weights [71]. This approach can help mitigate inter-sensor discrepancies by probabilistically minimizing uncertainty and leaning on the most reliable signal at any given moment.

In the tomato agrifood chain, this dual-weighting framework acts as a robust fail-safe spanning the entire production continuum. Under fluctuating greenhouse lighting, if visual sensors become blinded or highly uncertain, the D-S theory seamlessly shifts the decision-making reliance toward tactile, depth, or chemical sensors [68]. This holistic integration ensures a comprehensive, fault-tolerant assessment of tomato quality—from on-vine maturity monitoring to post-harvest defect sorting—while maintaining precise spatial localization to avoid mechanical bruising.

Despite its theoretical elegance, the primary limitation of this dual-weighting mechanism is its extreme computational intensity. The continuous calculation of uncertainty matrices and the execution of D-S combination rules impose a severe burden on edge processors. During rapid operations, this mathematical overhead can induce desynchronization between robotic perception and physical actuation, posing a direct risk to the agrifood chain. Future research must prioritize the development of lightweight, hardware-accelerated conflict resolution algorithms tailored for resource-constrained edge devices, ensuring a balance between decision-making accuracy and millisecond-level real-time performance.

### 4.3. Resource-Aware Processing and Edge Deployment

Currently, the evolution of agricultural embodied intelligence is increasingly driven by LLMs. Functioning as cognitive hubs, LLMs translate high-level semantic commands into zero-shot execution strategies, shifting agricultural robots from rule-based control to a semantics-driven autonomous operation [72]. However, the complexity of unstructured agricultural environments exposes the inherent limitations of text-vision perception. To achieve robust physical interaction, the field of robotic control is rapidly expanding toward multimodal large language models (MLLMs). By transforming heterogeneous signals into discrete tokens and aligning them within a unified embedding space via cross-modal attention, MLLMs construct a comprehensive semantic field that seamlessly integrates perception, reasoning and control [73]. Projecting this MLLM architecture into the agrifood chain presents a pathway for contact-rich manipulation. By natively fusing spatial visual data with a comprehensive spectrum of non-visual sensory tokens, agricultural end-effectors are endowed with multidimensional synergistic cognition. This framework enables adaptive reasoning and dynamic motion reconstruction during physical interactions. Harvesting robots can transcend conventional visual localization through hand–eye–nose coordination: leveraging vision for spatial alignment, tactile sensors for real-time elastic feedback and an electronic nose for absolute maturity detection. By autonomously aligning these heterogeneous inputs, the MLLM formulates precise agronomic decisions, allowing robots to precisely parse internal physiological states that remain inaccessible to purely optical sensors.

Despite the significant cognitive enhancements, the ultimate bottleneck for multimodal fusion in the agrifood chain lies in the deployment of these systems on edge computing hardware. Agricultural robots operating in the field are severely constrained by limited local computational resources and battery life. Processing high-dimensional data on localized edge boards frequently leads to jerky kinematics, critical decision-making delays and premature energy depletion. To circumvent these constraints, the academic community is converging on a holistic optimization strategy encompassing hardware, algorithms and system architecture:

1. Hardware-Level Event-Driven Perception: Conventional frame-based cameras exhaust power by continuously processing redundant static backgrounds. In contrast, bio-inspired neuromorphic sensors (event cameras) asynchronously capture only dynamic illumination changes. This event-driven approach drastically reduces data volume, motion blur, and power consumption, making it ideal for filtering out static foliage while tracking dynamic targets such as swaying tomato trusses during robotic manipulation or high-speed sorting [74].
2. Algorithm-Level Model Compression: To map massive multimodal networks onto edge devices, they must undergo rigorous structural compression. Techniques such as network pruning and knowledge distillation (KD) effectively condense the comprehensive feature-extraction capabilities of large teacher models into lightweight student architectures. For instance, optimized models like YOLOR-Slim have demonstrated that pruning and KD can slash computational loads to 1.9 GFLOPs and parameter counts to 1.4 M, enabling real-time, high-precision tomato main-stem detection and delicate pedicel localization on constrained edge devices with minimal inference latency [75].
3. System-Level Intelligent Offloading and Edge–Cloud Synergy: For high-dimensional tasks that inevitably exceed local processing limits, a systemic solution is required. Modern frameworks address this by combining adaptive learning algorithms with next-generation ultra-reliable low-latency communication networks [76]. By continuously evaluating environmental states and network conditions, reinforcement learning (RL) algorithms intelligently and dynamically offload heavy computational workloads to nearby edge servers or the cloud brain, achieving an optimal equilibrium between task latency and robotic energy efficiency.

Through this three-level optimization, a swarm of tomato robots can sustainably operate in remote, off-grid farmlands and seamlessly integrate with downstream processing facilities. By relying on low-power event cameras and compressed models for localized real-time navigation and non-destructive harvesting operations, the robots preserve their operational endurance. Simultaneously, complex analytical tasks are seamlessly offloaded via 6G networks to the cloud, ensuring high-precision operations without burdening the robot’s local edge board. During transmission, encryption guarantees that farmers’ proprietary data remains secure and inviolable.

Despite its immense potential, this edge–cloud continuum remains fragile in practical agricultural settings. The framework heavily relies on robust infrastructure, which is currently scarce in remote rural areas. Additionally, executing RL inference locally on edge nodes incurs non-trivial CPU overhead, while data encryption introduces latency penalties that conflict with the millisecond-level responsiveness required for robotic actuation. Future research must prioritize the integration of federated learning to enable decentralized, privacy-preserving model training. Furthermore, developing lightweight cryptographic alternatives, agricultural digital twins for preemptive resource allocation, and in-field energy harvesting mechanisms will be critical steps toward achieving truly autonomous and sustainable agricultural AI.

In summary, although isolated multisensory combinations have shown preliminary promise—such as robotic grippers integrating tactile and visual modules for fruit picking [32]—a comprehensive system-level validation across the entire agrifood chain remains unrealized. Consequently, a substantial TRL gap persists. While specific hardware modules have reached near-commercial stages (TRL 6–9), the proposed holistic architectures currently reside at TRL 3–5. In practical terms, this TRL 3–5 state indicates that these integrated frameworks are largely confined to analytical proofs-of-concept and isolated subsystem validations within controlled laboratory environments, serving primarily as conceptual blueprints rather than field-ready solutions [54]. Bridging this transition from theory to practice requires a concerted future effort focused on rigorous empirical validation. Crucially, by synergizing the proposed constraint-driven fusion strategies (i.e., scenario-driven mapping, readiness-based integration and resource-aware edge deployment) to tackle these practical bottlenecks, and adhering to a progressive developmental pathway that spans scenario-specific technological fine-tuning, empirical validation at localized nodes and full-scale field deployment, these conceptual paradigms will ultimately empower the intelligent evolution of the tomato agrifood chain.

## 5. Conclusions

The transition from vision-centric perception toward holistic multisensory perception marks a critical step in the intelligent management of the tomato agrifood chain. This perspective synthesizes the technological trajectory of optical sensing while elucidating the bottlenecks in environmental robustness and computational overhead that impede scalable deployment. To transcend these limitations, emerging physical (tactile and auditory) and chemical (olfactory and gustatory) modalities are established as indispensable dimensions for decoding internal physiological states inaccessible to optical sensors. A core contribution of this work is the conceptualization of the digital fingerprint, acting as a modeling bridge to deeply couple raw instrumental signals with subjective human sensory experiences. To address practical deployment challenges, a multimodal fusion architecture is proposed, incorporating scenario-driven mapping, readiness-based integration, and resource-aware processing to ensure resilient deployment in unstructured environments. These perception advancements provide a critical foundation for agricultural embodied intelligence and world models, fostering a transition toward intelligent labor, reshaping the tomato agrifood chain and offering a solution to mitigate food challenges and labor scarcity.

## Figures and Tables

**Table 1:** Typical mechanisms of tactile sensors and their comparative advantages and limitations.

Mechanism	Advantages	Limitations
piezoresistive	high sensitivity strong overload capacity	poor current stability of piezoresistors large volume high power consumption fragile contact surface
photoelectric	high spatial resolution low susceptibility to electromagnetic interference	low linearity under multi-force loading difficult calibration poor real-time performance
capacitive	large measurement range good linearity low manufacturing cost good real-time performance	large volume susceptible to noise interference poor stability
inductive	low manufacturing cost wide measurement range	difficult-to-control magnetic field distribution low resolution poor consistency across different contact points
piezoelectric	wide dynamic range durable	susceptible to thermal response effects

**Table 2:** Comprehensive comparison of multisensory perception for agricultural deployment.

Benchmarking Metrics	Vision	Tactile	Auditory	Olfactory	Gustatory
technology readiness level	8–9 (MV) 6–7 (SV)	6–7	5–6	3–5	3–5
accuracy	high	high	medium	high	high
response time	fast (MV) slow (SV)	fast	fast	medium	medium
throughput	high (MV) medium (SV)	low	high	low	low
calibration cost	low (MV) high (SV)	medium	low	high	high
maintenance requirements	low (MV) medium (SV)	medium	low	high	high
environmental robustness	low	high	low	low	low
cultivar robustness	medium (MV) low (SV)	high	high	low	low
economic feasibility	high (MV) low (SV)	medium	high	medium	relatively low
deployment barriers	canopy occlusion (MV) dynamic field illumination (MV&SV) computational overhead (SV) data interpretability (SV)	complex integration with end-effectors	ambient field noise interference	extreme environmental sensitivity	destructive sampling
key references	[50]	[51]	[33,40,41,42]	[52]	[53]

The abbreviations MV and SV denote machine vision and spectral vision, respectively. Technology readiness level (TRL) is assessed based on the standard 1–9 scale: TRL 1–3 represent basic research and proof of concept, TRL 4–5 involve validation in laboratory or controlled environments, TRL 6–7 involve prototype demonstration in relevant agricultural conditions, and TRL 8–9 indicate a fully commercialized system proven through successful real-world field operations [54]. Furthermore, based on data and judgments extracted from the reviewed literature regarding deployment realities, other qualitative ratings presented in the table were determined through comprehensive synthesis. Specifically, accuracy is categorized as high (>90%), medium (80–89%) or low (<80%) based on predictive/classification performance in relevant environments. Response time is defined as fast (<1 s; enabling real-time on-the-fly execution), medium (1–10 s; suitable for stationary or batch processing) or slow (>10 s; requiring prolonged acquisition or complex preprocessing). Throughput is high for non-contact and continuous processing with millisecond-level latency, medium for stationary inspection or serial processing, and low for batch processing requiring physical contact, significant signal stabilization time or sample extraction. Calibration cost and maintenance requirements are high if they require frequent expert-level recalibration or consumable replacement, medium if they require periodic standard or calibration maintenance and cleaning, and low for automated self-calibration or minimal maintenance. Environmental and cultivar robustness is high for stable performance with <10% accuracy degradation, medium for 10–20% degradation, and low for >20% degradation under varied conditions. Economic feasibility is defined as high for low-capital mass-market sensors, medium if they require professional equipment with moderate capital investment and specific system integration, low for highly capital-intensive laboratory-grade instrumentation, and relatively low if occupying an intermediate state between medium and low.

## Data Availability

Data sharing is not applicable.

## Abbreviations

The following abbreviations are used in this manuscript:

1D	One-Dimensional
2D	Two-Dimensional
3D	Three-Dimensional
3D-NDT	3D normal distributions transform
6G	Sixth generation
ANN	Artificial neural network
BAFusion	Bidirectional attention fusion
CASA	Computer-Assisted semi-automatic
CNN	Convolutional neural network
CP	Conformal prediction
DBSCAN	Density-Based spatial clustering of applications with noise
D-S	Dempster–Shafer
E-nose	Electronic nose
E-tongue	Electronic tongue
EGO	Efficient general ontology
ELM	Extreme learning machine
GFLOPs	Giga floating-point operations per second
GNN	Graph neural network
HSI	Hyperspectral imaging
HS-SPME-GC-MS/MS	Headspace solid-phase microextraction-gas chromatography–tandem mass Spectrometry
ISO	International organization for standardization
KD	Knowledge distillation
kNN	k-Nearest neighbors
LDA	Linear discriminant analysis
LLM	Large language model
MLLM	Multimodal large language model
MOS	Metal oxide semiconductor
MV	Machine vision
NIR	Near-Infrared
RGB	Red–Green–Blue
RGB-NIR	Red–Green–Blue near-infrared
RL	Reinforcement learning
RMSEP	Root mean square error of prediction
*R_p_*^2^	Prediction determination coefficient
*R*^2^	Coefficient of determination
RPD	Residual predictive deviation
RT-DETR	Real-Time detection transformer
SERS	Surface-Enhanced Raman spectroscopy
SegLoRA	Segmentation low-rank adaptation
SV	Spectral vision
SVM	Support vector machine
Swin	Shifted window
SWIR	Short-Wave-Infrared
TCARI	Transformed chlorophyll absorption in reflectance index
ToF	Time of flight
TRL	Technology readiness level
Vis-NIR	Visible-Near-Infrared
